# Vancomycin containing PLLA/β-TCP controls experimental osteomyelitis *in vivo*

**DOI:** 10.1186/s13018-014-0114-3

**Published:** 2014-11-19

**Authors:** Berna Kankilic, Elif Bilgic, Petek Korkusuz, Feza Korkusuz

**Affiliations:** Department of Biotechnology, Institute of Applied Sciences, Middle East Technical University, Çankaya, Ankara 06800 Turkey; Department of Histology and Embryology, Hacettepe University Faculty of Medicine, Sihhiye, Ankara, 06100 Turkey; Department of Sports Medicine, Hacettepe University Faculty of Medicine, Sihhiye, Ankara, 06100 Turkey

**Keywords:** Bone, Osteomyelitis, Drug delivery, Rat, Vancomycin, PLLA, β-TCP, MRSA, Infection

## Abstract

**Background:**

Implant-related osteomyelitis (IRO) is recently controlled with local antibiotic delivery systems to overcome conventional therapy disadvantages. *In vivo* evaluation of such systems is however too little.

**Questions/purposes:**

We asked whether vancomycin (V)-containing poly-l-lactic acid/β-tricalcium phosphate (PLLA/β-TCP) composites control experimental IRO and promote bone healing *in vivo*.

**Methods:**

Fifty-six rats were distributed to five groups in this longitudinal controlled study. Experimental IRO was established at tibiae by injecting methicillin-resistant *Staphylococcus aureus* (MRSA) suspensions with titanium particles in 32 rats. Vancomycin-free PLLA/β-TCP composites were implanted into the normal and infected tibiae, whereas V-PLLA/β-TCP composites and coated (C)-V-PLLA/β-TCP composites were implanted into IRO sites. Sham-operated tibiae established the control group. Radiological and histological scores were quantified with microbiological findings on weeks 1 and 6.

**Results:**

IRO is resolved in the CV- and the V-PLLA/β-TCP groups but not in the PLLA/β-TCP group. MRSA was not isolated in the CV- and the V-PLLA/β-TCP groups at all times whereas the bacteria were present in the PLLA/β-TCP group. Radiological signs secondary to infection are improved from 10.9 ± 0.9 to 3.0 ± 0.3 in the V-PLLA/β-TCP group but remained constant in the PLLA/β-TCP group. Histology scores are improved from 24.7 ± 6.5 to 17.6 ± 4.8 and from 27.6 ± 7.9 to 32.4 ± 8.9 in the CV-PLLA/β-TCP and the V-PLLA/β-TCP groups, respectively. New bone was formed in all the PLLA/β-TCP group at weeks 1 and 6.

**Conclusions:**

CV- and V-PLLA/β-TCP composites controlled experimental IRO and promoted bone healing.

**Clinical relevance:**

CV- and V-PLLA/β-TCP composites have the potential of controlling experimental IRO and promoting bone healing.

## Introduction

Implant-related osteomyelitis (IRO) [1] is a complex bone infection that requires the surgical removal of sequester or implant [2] followed with the debridement of the infected tissues [3] and high dosage antibiotic regimen for 6 weeks [4]. *Staphylococcus aureus* is the main causative organism for osteomyelitis [5]. It can form a biofilm, an extracellular polymeric matrix, in which cells can communicate and protect themselves from antibacterial agents [6]. Methicillin-resistant *S. aureus* (MRSA) is a strain of *S. aureus*, which mainly caused nosocomial bone infections [7]. Vancomycin is a frequently used antibiotic against infections caused by MRSA [8]. Local antibiotic delivery systems [9,10] are proposed to control IRO safely and efficiently as parenteral administration of high doses of vancomycin for long durations may lead to some effects [11].

Although various degradable polymers [12,13] and β-TCP [14] were used to release antibiotics, *in vivo* evaluation of these systems was rarely [15,16] undertaken. A vancomycin-containing poly-l-lactic acid/β-tricalcium phosphate (V-PLLA/β-TCP) composite was recently developed and characterized for this reason [17]. PLLA slowed vancomycin release and β-TCP aided mesenchymal stem and Saos-2 bone cells attachment, proliferation, and differentiation *in vitro* [17]. This composite was further dip coated (C) with PLLA and 63.1% and 91.9% of the vancomycin released on days 1 and 42, respectively. CV- and V-PLLA/β-TCP composites furthermore generated the extracellular bone matrix and attained mineralization that may point out tissue regeneration [17]. In this study, we asked whether CV- and V-PLLA/β-TCP composites controlled experimental IRO and promoted bone healing *in vivo*.

## Materials and methods

**Figure 1:**
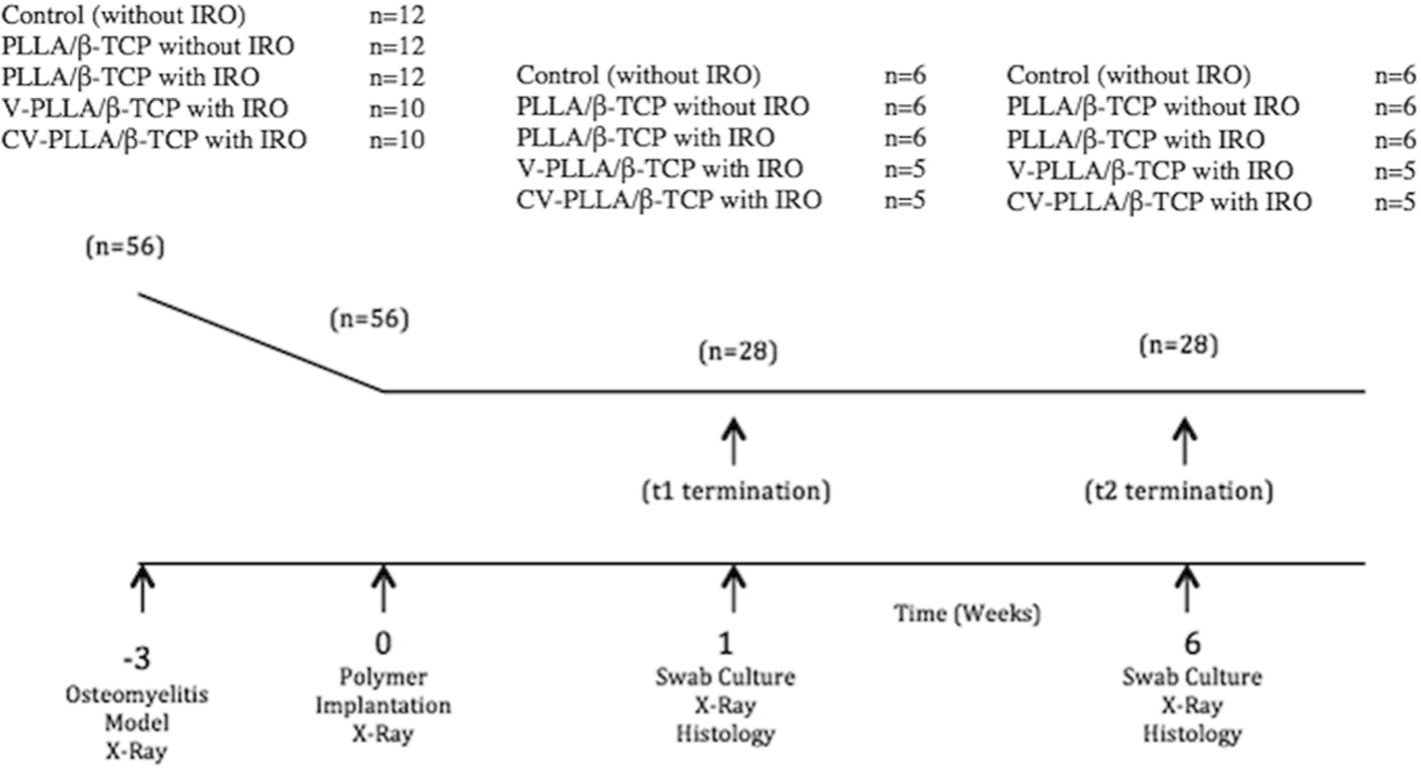
**The experimental design and study groups are shown.** *n* = number; *t* = time; IRO = implant-related chronic osteomyelitis.

A longitudinal controlled study in rat tibiae was designed after ethical committee approval. Independent variables were groups (*n* =5) and time (*n* =2) and dependent variables were radiological, histological, and microbiological scores. Free online software was used to calculate the sample size of the study. The power 1-beta and type I error rate alpha were set to 0.8% and 5%, respectively. Power analysis with 56 rats revealed 0.81% confidence; therefore, minimum 10 rats were assigned into one of the five experimental groups. In the control group, the tibiae of the rats underwent sham operation without IRO establishment (*n* =12). PLLA/β-TCP composites were implanted into the non-infected tibiae in the second group (*n* =12). The PLLA/β-TCP, the V-PLLA/β-TCP, and the CV-PLLA/β-TCP composites were implanted into the IRO-established tibiae (*n* =12, *n* =10, and *n* =10), respectively, in the last three groups (Figure 1). We examined the groups after 1 and 6 weeks and quantified their radiological, histological, and microbiological findings.

Composites were developed and characterized as previously described [17]. Briefly, 8% vancomycin solution was prepared with 920 mg deionized water. This solution was mixed with 720 mg β-TCP and dried at 37°C for 12 h. The final mixture was added to 3% PLLA solution that contained 2.67 g chloroform and molded to obtain a spherical shape. MRSA (strain: ATCC 2592) was obtained from the microbiological collection of Ankara Numune Hospital Microbiology Laboratory. We used 3-month-old male Sprague–Dawley rats weighing 300–350 g housed in a temperature-controlled room (22°C–24°C) with 12-h light and 12-h dark cycles. Rats were given free access to water and food without antibiotics for 24 h a day. They were anesthetized by intraperitoneal 0.2 mL ketamine chloride (Ketasol 10%, Richter-Pharma, Wels, Austria) and 0.1 mL xylazine (Alfazyne 2%, Alfasan, Woerden, the Netherlands) injection. The left hind limb was shaved and cleaned with iodine solution (Polyod, Drogsan, Ankara, Turkey). The proximal anterior cortex of the tibia was exposed and drilled through with a 3.2-mm burr (Dremel, Gerlingen, Germany). We irrigated the burr hole with 0.09% sodium chloride solution (Eczacıbaşı-Baxter, Istanbul, Turkey) and injected a 0.1 mL MRSA suspension (1 × 10^6^ CFU/cm^3^) into the intramedullary cavity with approximately 200 1.0 × 1.0-mm titanium particles to establish IRO in the last three groups. The burr hole was closed with bone wax (Ethicon, Johnson & Johnson, New Brunswick, NJ, USA) to prevent MRSA from leaking into the subcutaneous soft tissues. A sham operation was undertaken in the control group. Only the tibia was exposed and no other intervention was taken place. For the PLLA/β-TCP implantation, the tibiae were drilled and no MRSA suspension was given. The skin and subcutaneous tissues were sutured with 2.0 silk (Doğsan, Trabzon, Turkey), and closure was cleaned and sprayed with an antibacterial film (Opsite, Smith & Nephew, London, England) [18]. Rats were put into cages allowing their free movement and fed with regular diet. Anterior-posterior and lateral radiographs of the tibia were obtained after 3 weeks in the IRO groups using the Siemens Multix-C (Siemens AG, Erlangen, Germany) X-ray device at a setting of 46 kV and 2.5 mAs/s to confirm radiological signs of IRO establishment. The distance between the X-ray source and the samples was 100.0 cm at examination. Agfa Crurix X-ray films (Agfa, Mortsel, Belgium) and the Crurix 60 (Agfa, Mortsel, Belgium) automatic developing machine were used. When macroscopic and radiologic signs of IRO became definite, all closures were reopened and only one composite implanted into the burr hole was found in the tibia based on its group. After 1 and 6 weeks, radiographs were obtained and two orthopedic surgeons who had no prior information of the groups independently evaluated the radiographs for the presence of IRO. Radiologic criteria were: (1) periosteal reaction, (2) diaphyseal widening, (3) osteolysis, (4) bone deformation, (5) sequestrum formation, (6) joint effusion, and (7) soft tissue swelling. Parameters 1 to 4 were graded as (0) absent, (1) mild, (2) moderate, and (3) severe, and parameters 5 to 7 were graded as (0) absent and (1) present [19]. Swab cultures were obtained for microbiological analysis. A sterile stripe was smeared into the implantation site to take culture. The stripes were streaked onto blood agar plates and incubated at 37°C for 12 h and the presence or absence of growth in the media was recorded. The animals were sacrificed by lethal doses of anesthetics and tibiae of the left hind leg were dissected. All soft tissues were removed and bones were fixed in 10% phosphate buffered formalin (pH 7.0) at room temperature for histological analysis at termination. Samples were then decalcified by immersion in De Castro solution [nitric acid (Merck, Darmstadt, Germany), chloral hydrate (Merck, Darmstadt, Germany), and distilled water] for 5–10 days before dehydration. Tibiae were rinsed in buffer and dehydrated in a graded series of ethanol before embedding in paraffin in an automated tissue processor (TP-1020, Leica, Wetzlar, Germany). For each tibia, we determined the severity of infection with 60–80 5-μm-thick hematoxylin and eosin and trichrome-stained serial sections using a DMR 6000 microscope equipped with the DC500 digital camera (Leica, Wetzlar, Germany). Semi-quantitative image analysis was undertaken using the Qwin Plus computer image analysis system (Leica, Wetzlar, Germany). The medullar cavity (leukocytes, microabscesses, granulation tissue, fibrosis), cortex (destruction of the cortex, enlarged Haversian canals, leukocytes, microabscesses, granulation tissue, fibrosis), and periosteal reaction (quantity) were scored according to the literature [according to severity as none, slight, moderate, or much from 0 to 4] in at least ten regions of interest [20]. The total score was calculated and noted for the statistical analysis.

Descriptive values are expressed as the minimum, the maximum, and the median. As all the scores for the control group were zero, this group was taken off from statistical analysis. Normality of distribution and homogeneity of variances were adjusted using the Shapiro-Wilk test. Radiological and the histological scores were analyzed by nonparametric tests (Kruskal-Wallis for multiple comparison and Mann Whitney U as *post hoc* test with Bonferroni correction; *p* <0.0083 was considered significant.). Spearman’s test was used to assess correlations between continuous radiology and histology variables. The Pearson product–moment correlation coefficient test was used for the interobserver and intraobserver reliabilities.

Each author certifies that Hacettepe University Animal Care and Use Ethics Committee approved (no. 2007/65-1) the animal protocol for this investigation and that all investigations were conducted in conformity with ethical principles of research.

## Results

**Figure 2:**
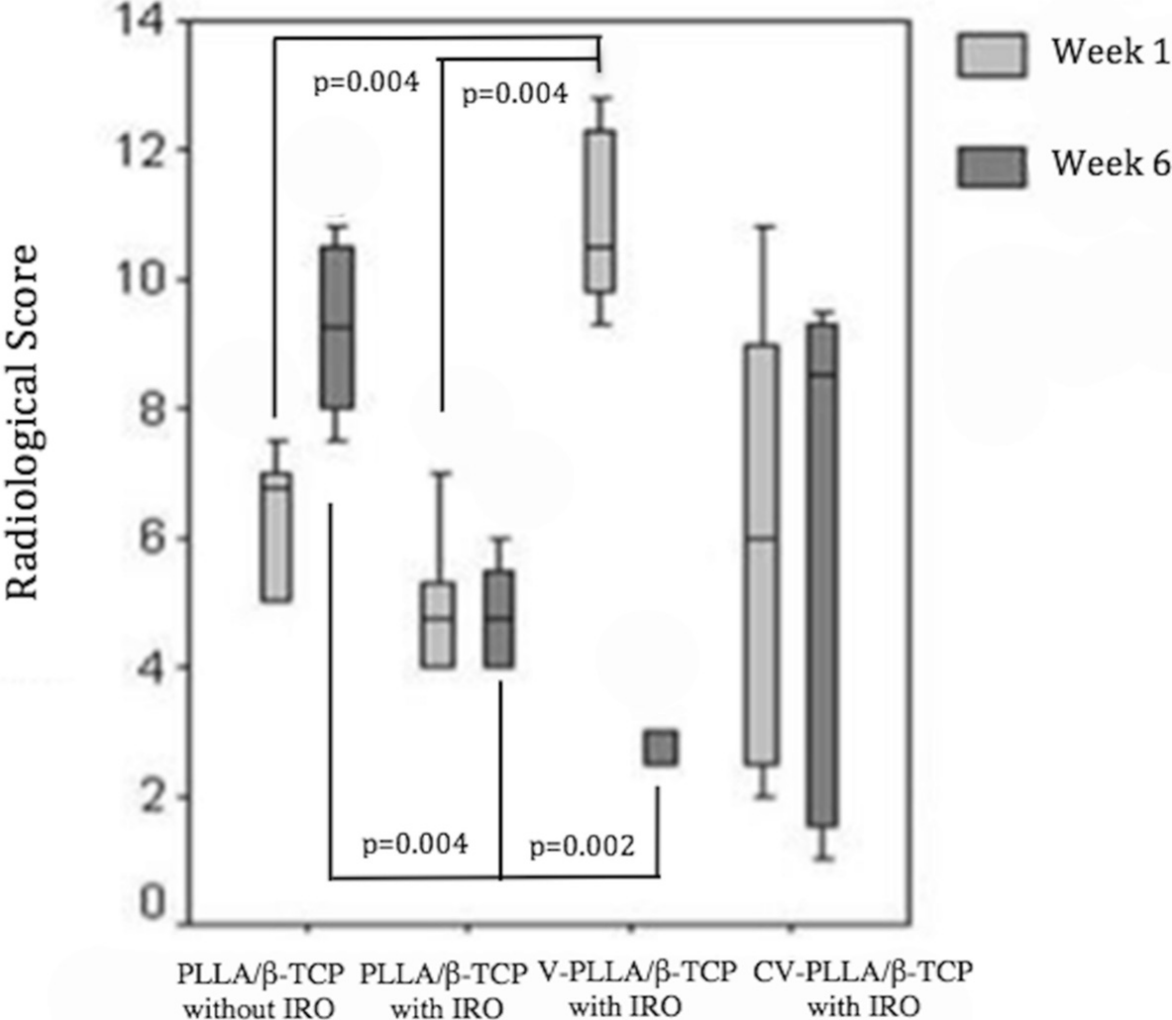
**The descriptive data of radiological scores of the groups are shown for weeks 1 and 6**: **(minimum, maximum, and median values).** V-PLLA/β-TCP with IRO score recruited and PLLA/β-TCP without IRO score regressed on week 6. Note that *p* <0.0083 for a-c, b-c, d-e, d-f.

MRSA did not grow in the CV- and V-PLLA/β-TCP groups at any time. MRSA and *Escherichia coli* were isolated from four and two of six, and from three and three of six tibiae in the PLLA/β-TCP group at week 1 and 6, respectively. Radiological score of the V-PLLA/β-TCP group improved (*p* =0.043) at week 6 (Figure 2). Interobserver and intraobserver reliabilities (*r*) for the presence or absence of infection were 0.95 and 0.98, respectively. The radiographic scores correlated with histological scores at weeks 1 (*r* =0.896; *p* <0.05) and 6 (*r* =0.90; *p* <0.05). Microbiological and radiological scores from this *in vivo* study suggest that IRO was controlled in the CV- and V-PLLA/β-TCP groups but not in the PLLA/β-TCP group.

**Figure 3:**
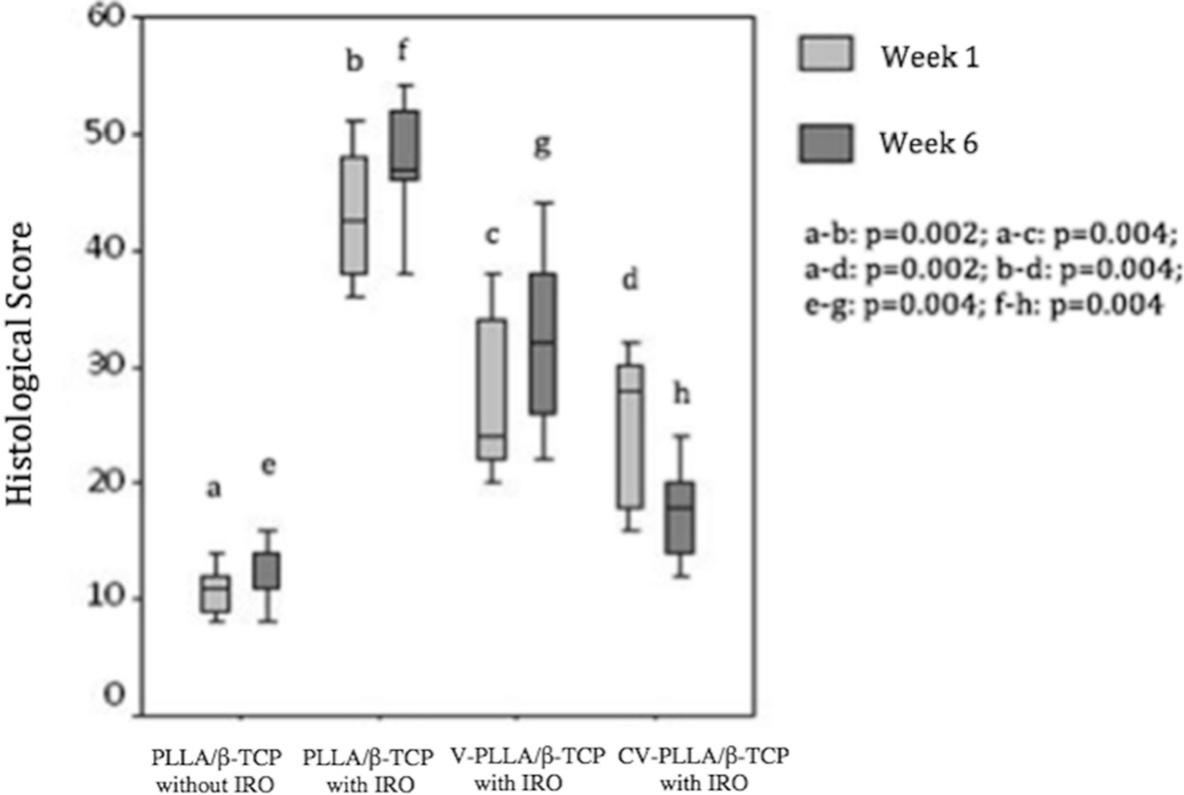
**The descriptive data of histological scores of the groups are shown for weeks 1 and 6**: **(minimum, maximum, and median values).** PLLA/β-TCP without IRO exhibited better scores than the other groups at weeks 1 and 6. V-PLLA/β-TCP and CV-PLLA/β-TCP presented better scores compared with that of PLLA/β-TCP with IRO at week 6. Note that *p* <0.0083 for a-b, a-c, a-d, e-f, e-g, e-h.

**Figure 4:**
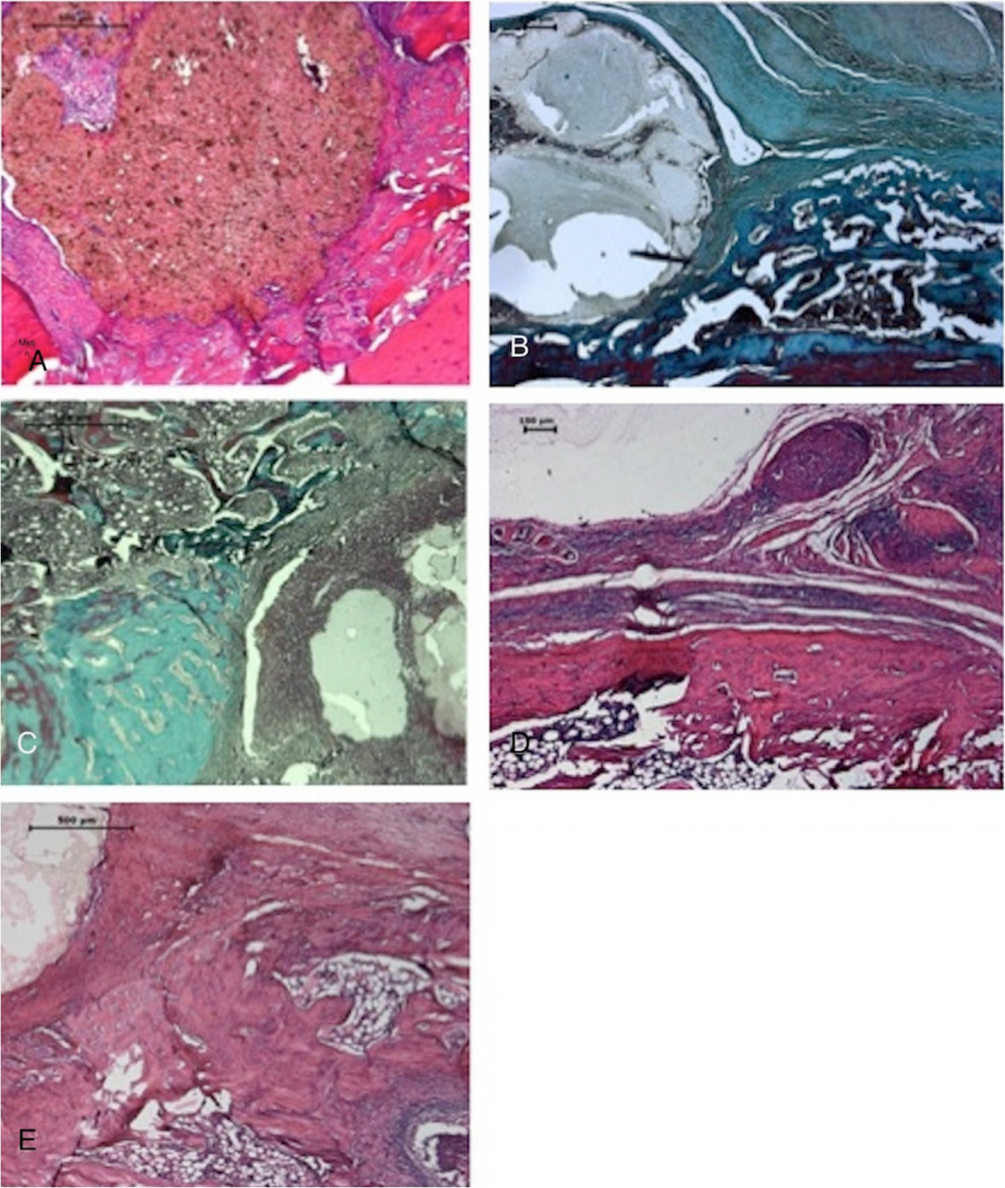
**Histological images** **(A-E).** PLLA/β-TCP without IRO group had a mild tissue reaction in **(A)** week 1 with HE **(B)** and week 6 with MT. The implant is degrading but still present at 6 weeks. PLLA/β-TCP with IRO group had evident bone damage and granulation tissue formation with mononuclear cell infiltration and periosteal reaction in **(C)** week 1 with MT and **(D)** week 6 with HE, respectively. V-PLLA/β-TCP had better morphologic features of the bone and marrow in **(E)** week 1 with HE with the presence of new forming healthy spongy bone. *CT* connective tissue, *TB* trabecular bone, *CB* compact bone, *BM* bone marrow, *GT* granulation tissue, *I* implant, *HE* hematoxylin and eosin, *MT* Masson’s trichrome.

PLLA/β-TCP composites implanted into normal bone without IRO presented healthy cortical osteons and minimal or no periosteal reaction histologically, receiving better scores than the other groups at week 1 (Figure 3). PLLA/β-TCP composites with IRO exhibited cortical bone damage with enlarged Haversian canals and granulation tissue formation. Mononuclear cells infiltrated the medullary canal in the same group. Broad periosteal reaction at weeks 1 and 6 were observed in the PLLA/β-TCP groups with IRO. The CV-PLLA/β-TCP group had comparatively better (*p* =0.004) histological scores compared with that of the PLLA/β-TCP group at week 6 (Figure 4).

## Discussion

Staphylococcal bacteria have adhesion potential to biomaterials [21] resulting in chronic osteomyelitis and IRO [6]. This bone infection can be controlled with local drug delivery systems. CV- and V-PLLA/β-TCP composites can be a choice since remission of IRO was achieved with these implants. This study had some limitations. *E. coli* was isolated in three PLLA/β-TCP composites, which indicated surgical site contamination. Complex bacterial contamination is a frequent state in osteomyelitis due to the open wounds and/or multiple surgeries [22]; however, CV- and V-PLLA/β-TCP composites were able to overcome this complication. *In vivo* degradation time and reduction of mechanical strength of our material were not measured, as eradication of MRSA was our primary aim.

Microbiologic findings revealed control of the infection in the CV- and V-PLLA/β-TCP groups. Bacteria did not grow in these groups at any time. Radiological scores correlated well with histological scores and improvement was achieved in the CV- and V-PLLA/β-TCP groups. These findings were in line with previous studies [23,24]. The radiologic score of the PLLA/β-TCP group without IRO group deteriorated slightly at week 6 most probably due to composite degradation [25]. A decrease in pH owing to PLLA degradation could have caused bone resorption [26] in this group. Histological findings of the CV- and V-PLLA/β-TCP groups presented favorable biocompatibility. TCP is an osteoconductive material [27] and influences bone ingrowth in the pores of the composite. This finding was in line with another study [16] that demonstrated new trabecular bone formation at the defect margins in biodegradable TCP composites. Quantitative histology revealed biocompatibility and osteointegration as in another study [28].

## Conclusion

In conclusion, vancomycin-containing PLLA/β-TCP composites presented favorable radiological, histological, and microbiological results of remission and according to clinical relevance of the study, they can be used to control IRO in the near future.
